# The Awareness of Risks Associated with OTC Drugs Available in Non-Pharmacy Outlets among Polish Patients—A Cross-Sectional Study

**DOI:** 10.3390/healthcare9020187

**Published:** 2021-02-09

**Authors:** Jacek Dulęba, Urszula Religioni, Emilia Słodka, Andrzej Fal, Jerzy Krysiński, Piotr Merks

**Affiliations:** 1 Collegium Medicum in Bydgoszcz, Nicolas Copernicus University, 85-067 Torun, Poland; jac.duleba@gmail.com (J.D.); emilia.ula@gmail.com (E.S.); 2 Warsaw School of Economics, 02-568 Warsaw, Poland; urszula.religioni@gmail.com; 3 Collegium Medicum, Cardinal Wyszyński University, 01-815 Warsaw, Poland; a.fal@uksw.edu.pl; 4 Department of Pharmaceutical Technology, Collegium Medicum in Bydgoszcz, Nicolas Copernicus University, 85-067 Torun, Poland; kiztechpostlek@cm.umk.pl

**Keywords:** OTC drugs, non-pharmacy outlets, community pharmacy, pharmacist, Poland

## Abstract

Background: Many OTC drugs are available in non-pharmacy outlets, and as such the risk of overuse and/or improper use of this class of drugs is more likely. In light of this observation, our study was conducted with the aim of exploring opinions on non-pharmaceutical distribution among Polish patients. This study was a part of an informative project to educate patients about the possible risks associated with the use of OTC medicines. Methods: We conducted a cross-sectional study among Polish patients in which we used an authorial questionnaire, previously tested via content, face validity, and a pilot study. The questionnaire was distributed both electronically and in a traditional form. Results: ‘Time saving’ had a statistically significant role in the patient’s decision about purchasing drugs in a non-pharmacy outlet (*p* = 0.0052; R = 0.276994). The lack of a pharmacist’s consultation/advice (*p* = 0.0072; R = −0.3290490), improper storage (*p* = 0.0044; R = −0.238246), risk of overdose (*p* = 0.0125; R = −0.189564), and the risk of purchasing out-of-date drugs (*p* = 0.0125; R = −0.145411), as well as the risk of purchasing falsified drugs (*p* = 0.0265; R = −0.159428), were all associated with the patient’s non-willingness to purchase drugs in non-pharmacy outlets. Patients supported the idea of the elimination of non-pharmacy outlet distribution (*p* = 0.0074; R = −0.195932); they also emphasized that they are advocates of purchasing drugs only in community pharmacies (*p* = 0.0006; R = −0.238625). Conclusion: Patients are aware of the risks associated with buying drugs outside of community pharmacies. They perceived pharmacists as professional health care advisors and supported the fact that OTC drugs should be available only via pharmaceutical distribution.

## 1. Introduction

Today, we can observe a constant growth of the self-medication phenomenon among patients, which could be associated with the increased number of drugs that are available without a prescription [1]. Undoubtedly, self-medication does have some advantages, such as getting patients more involved in their treatment, which leads to an increase in the proper use of medications [2]. On the other hand, self-medication can lead to adverse events, exacerbate symptoms, and, consequently, worsen prognoses, particularly among chronically ill patients and the elderly [3]. Moreover, the overuse of OTC drugs can be harmful, especially when these products are dispensed without professional advice and when there are some doubts associated with the conditions in which the drugs were stored [4,5]. It should be highlighted that the lack of professional advice and doubtful storage conditions are more common in non-pharmacy outlets [6]. Pharmacists could play an important role in preventing drug-related problems associated with OTC drugs and decrease the possibility of adverse events and drug–drug interactions [7]. This kind of supervision is not possible with non-pharmaceutical distribution in outlets.

In light of these observations, some studies support the hypothesis that a community pharmacy is the only place where patients can receive professional drug information and be certain about proper drug storage [8,9,10]. Merks et al. proved that high-quality pharmaceutical services are important for both British and Polish patients while purchasing drugs in a community pharmacy [1]. Studies have shown that drug distribution in non-pharmacy outlets is linked with many potential risks, such as the use of various medicinal products that all contain the same active ingredient which can result in overdosing or duplication of therapy [2].

In Poland, distribution via non-pharmacy outlets is widespread [3,4]. Polish pharmaceutical law specifies that only OTC drugs which have been available on the market for at least 5 years in the OTC category can be dispensed in a non-pharmacy distribution [5]. However, the list of drugs available in non-pharmacy outlets in Poland is quite long, particularly if we compare this list with other European countries. In Germany and Austria, only natural drugs are accessible outside of the community pharmacy. In France, all drugs are available only through a community pharmacy [6]. Lind et al. reported that Swedish legislation is more strict than British law, and limits the number of OTC drugs available in non-pharmacy outlets. This could be associated with the fact that Sweden is now in a different phase of the deregulation effort [7].

In the light of the above-mentioned observations, it seems useful to know the perspective of Polish patients on the use of OTC drugs purchased in non-pharmacy outlets. The aim of our project was to investigate the opinions of Polish patients about drug distribution in non-pharmacy outlets with special attention to patients diagnosed with cardiovascular diseases. This study was a part of an informative project to educate patients about the possible risks associated with the use of OTC medicines. Our goal was also to emphasize the role of pharmacists in providing information about medicines, which can significantly influence the more appropriate use of these products among patients.

## 2. Material and Methods

### 2.1. Settings

Our study is cross-sectional and based on a quantitative approach. In 2017, a questionnaire was distributed among individuals who participated in an open university lecture—‘Medical Wednesday’—an academic initiative dedicated to improving patient knowledge about different diseases and health-related matters in various medical disciplines. We provided a questionnaire upon room entry and collected it on leaving. We also collected questionnaires from a dermatological outpatient clinic. Finally, we used an electronic platform, a free-of-charge tool that specializes in collecting questionnaires and enabling continuous analysis of data (descriptive statistics). This tool prevented collecting information from a particular IP address more than once. A link to the questionnaire was distributed via Facebook and Google Mail among individual respondents (convenient sampling) and also placed on professional sites associated with health-related matters, e.g., promoting preventive behavior in patients diagnosed with chronic diseases. The individuals who distributed the questionnaires were trained pharmacy students at an undergraduate level. The inclusion criterion required respondents to be older than 18 years of age. To summarize, we collected a total of 400 questionnaires including 250 in the paper version.

### 2.2. Survey Instrument

The proper research tool was an authorial questionnaire. The questionnaire was prepared after an extensive search of the literature and based on the experiences of members of the research team. It was based on the Likert scale and questions were composed of both single and multiple choice. The first part of the questionnaire was aimed at collecting sociodemographic data: gender, age category and place of residence. This served as a point of reference for subsequent dependences. Additionally, the patient was asked about any accompanying cardiovascular diseases. In the case of a positive response, the patient was asked to answer about issues related to these diseases (diagnosed by a doctor), subsequent hospitalizations and any drugs used in the past three months. The second part of the questionnaire was about knowledge of the concept of an OTC drug, the frequency of buying drugs of this category, and the place other than a pharmacy in which the medication had been purchased. The aim was to research the most common pharmacy and non-pharmacy OTC needs. Patients were asked about the reasons for purchasing drugs outside of a pharmacy and about their awareness of the risks of direct drug use from non-pharmacy outlets. We focused on the dangers of changes in the pharmacokinetic and pharmacodynamics of drugs caused by improper storage, or the increased risk of overdosing. Additionally, we verified patients’ trust in pharmacist, which would be indicated by, for example, asking the pharmacist to consult any drug purchasing. The third part of the questionnaire was of the risks of interactions between OTC drugs and drugs commonly used in cardiovascular disorders. For the patient’s convenience, the brand names of preparations available in pharmacies were given instead of the international non-proprietary names of drugs, and the symptoms which had possibly occurred after use were mentioned. Awareness of the risks of OTC drug overdose has been studied. As in the case of interactions, the drug’s trade names and the characteristic symptoms of overdose were described. Patients were additionally asked to comment on proposed solutions to reduce the risks of non-pharmacological OTC drugs. The questionnaire was then tested in a pilot analysis among ten adult respondents.

### 2.3. Data Analysis

Descriptive statistics were used to summarize the data. Multi-level logistic regression analysis was applied to investigate the associations between the patient’s willingness to purchase OTC drugs in non-outlet distribution and sociodemographic characteristics as well as different factors mentioned in the questionnaire, e.g., the price of the drug, saving time, or consultation with a pharmacist. Multi-level ordered logistic regression is often used when the dependent variable is discreteness and may take more values than in a binary model—yes/no; therefore, this statistical method was suitable for our study. A p-value below 0.05 was considered statistically significant.

### 2.4. Ethics

No ethical dilemma was associated with our study. Prior to beginning our study, we received positive feedback from the Ethical Commission at the Collegium Medicum in Bydgoszcz, Poland, which is part of the Nicolaus University in Toruń, Poland, specialized in training health care professionals. All participants were informed about the aim of the study and were informed that they could withdraw their consent at any time before the end of study.

## 3. Results

Out of 400 respondents, 275 (68.75%) were female. The largest number of patients who participated in the study was between the ages of 18 and 30—58.25% of respondents—while patients who were at least 70 years of age constituted only 3.00% of the respondents. As a place of residence, participants most frequently indicated cities with 100–500 thousand inhabitants—40.25%. In our study, 20% of patients had cardiovascular disease, most commonly with hypertension, diabetes, lipid disorders and arthritis. Bisoprolol (Bisocard) and perindopril (Prestarium) were the most frequently prescribed drugs; however, patients also used metformin (Siofor), simvastatin (Simvacard), captopril (Captopril), amlodipine (Amlozek) and gliclazide (Diaprel). The characteristics of the study population are presented in Table 1. A total of 53.75% of respondents were not familiar with the term ‘OTC drugs’, requiring an explanation of what OTC drugs are. OTC drugs were most frequently purchased by the patients less than once a month ago—61.50%. Apart from community pharmacies, patients bought OTC drugs in supermarkets, grocery stores, and drugstores. A large number of respondents did not buy drugs in non-pharmacy outlets.

‘Time saving’ had a statistically significant role in the patient’s decision about purchasing drugs in a non-pharmacy outlet (*p* = 0.0052; R = 0.276994). The lack of pharmacists’ consultation/advice (*p* = 0.0072; R = −0.3290490), improper storage (*p* = 0.0044; R = −0.238246), risk of overdose (*p* = 0.0125; R = −0.189564), and the risk of purchasing out-of-date drugs (*p* = 0.0125; R = −0.145411), as well as the risk of purchasing falsified drugs (*p* = 0.0265; R = −0.159428), were all associated with the patient’s low intensity of purchasing OTC drugs outside the pharmacy. Consultations with a pharmacist were deemed the most important in the context of drug–food interactions (*p* = 0.0245; R = 0.190319). For patients, only the interaction between aspirin and perindopril was almost statistically significant (*p* = 0.0582; R = −0.199522). Patients supported the idea of the elimination of non-pharmacy outlet distribution (*p* = 0.0074; R = −0.195932); they also emphasized that they are advocates of purchasing drugs only in community pharmacies (*p* = 0.0006; R = −0.238625). The data is shown in Table 2.

## 4. Discussion

Our research confirmed that patients prefer drug distribution in the community pharmacy more than non-pharmacy outlets, which is related to the intensity of purchases. Moreover, patients recognized many dangers which may be associated with buying drugs in non-pharmacy outlets, among others, improper storage and the risk of providing falsified drugs. However, patients only partially noticed the role a pharmacist plays in the supervision of drug distribution, which could be associated with the fact that only drug–food interactions were considered important from the patient’s perspective. The most alarming result was that 31% of patients used a combination of two or more OTC drugs at the same time, and were not aware of any potentially harmful interactions between the OTC drugs.

Still, there are limited studies aimed at exploring the patient’s perspective on non-pharmacy outlets. Our findings are consistent with a recently published article from Sweden, written by Westerlund et al., which confirmed that Swedish patients consider the community pharmacy as the most appropriate place to buy OTC drugs. Moreover, Swedish patients revealed that a pharmacist’s consultations and trust in pharmacists’ professionalism are the most important factors in their decision about buying OTC drugs in a community pharmacy [8]. Australian patients displayed trust in pharmaceutical advice [9], and, for Flemish patients, pharmacists are the primary source of professional information about OTC drugs [10]. Interesting findings can also be seen in the study published by Villako et al. in which patients under 40 years of age were less willing to consult with physicians about OTC drugs. In the same study, we can find information that advising the patient about drugs was less important for patients with only an elementary school education [11]. Pharmaceutical advice is important due to the risks associated with self-treatment. In light of this observation, a German study revealed that 44.5% of patients did not read the drug leaflet before using an OTC drug [12]. Recently, due to amendments made to regulations, more and more Rx drugs are now available as OTC drugs; thus, the necessity of professional supervision over self-treatment is even more crucial than it was a decade ago [13]. In the context of Poland, Piecuch et al. noted that pharmacists recognized how important drug counseling is for patients during self-treatment and deciding whether medications should be taken [14]. However, some pharmacists are still not confident in providing pharmaceutical services, predominantly due to their lack of knowledge (self-declared opinions) [15]. Improving communication between pharmacists and patients could be considered a potential solution, as highlighted by Hungarian scientists in their research aimed at exploring patients’ opinions on OTC drugs [16].

Our study has several limitations. First of all, the study was a part of an educational project, and the questions in the questionnaire could be suggestive, indicating a risk of medication misuse. Secondly, we investigated only a select group of individuals, mostly young people who do not need multiple medications compared to the geriatric population. Also, the younger population may have different shopping habits. Thus, our findings may not be representative of the opinions of the whole Polish population. As such, further representative studies are warranted. Moreover, there is a strong need to conduct qualitative studies aimed at exploring patients’ opinions in a more in-depth way, which is not possible using only a quantitative approach. Due to the limited number of respondents who confirmed that they had cardiovascular diseases diagnosed by a doctor, analysis in this subpopulation was not possible. In the future, studies on the risk of OTC drugs involving only the group of patients with cardiac diseases may prove important.

## 5. Conclusions

Patients are aware of the risks associated with buying drugs outside of community pharmacies. They perceived pharmacists as professional health care advisors and supported the fact that OTC drugs should be available only via pharmaceutical distribution. Nevertheless, further representative studies are needed to prove this issue.

## Figures and Tables

**Table 1:** Characteristics of the study population.

	Sum (n = 400)
Gender	
Female	275 (68.75%)
Men	125 (31.25%)
Age	
18–30	233 (58.25%)
31–40	40 (10.00%)
41–50	36 (9.00%)
51–60	39 (9.75%)
61–70	40 (10.00%)
Over 70	12 (3.00%)
Place of residence	
Village	73 (18.25%)
City < 10 thousand.	24 (6.00%)
City 10–50 thousand	57 (14.25%)
City 50–100 thousand	53 (13.25)
City 100–500 thousand	161 (40.25%)
City > 500 thousand	32 (8.00%)
Diseases of the cardiovascular system	
Yes	80 (20.00%)
No	320 (80.00%)
	**Sum (n = 80)**
Cardiovascular disease	
Hypertension	55
Heart failure	4
Arrhythmia	15
After myocardial infarction	2
Diabetes	19
Lipid disorders	16
Atherosclerosis	9
After stroke	1
Congenital heart defect	4
Time of hospitalization	
3 months ago	3 (3.75%)
6 months ago	1 (1.25%)
9 months ago	1 (1.25%)
12 months ago	5 (6.25%)
More than 12 months ago	13 (16.25%)
No	51 (63.75%)
I don’t remember	6 (7.50%)
I don’t understand the question	0 (0.00%)
Medications used in cardiovascular disease	
Amlozek (amlodipine)	7
Captopril	8
Xartan (losartan)	2
Lacipil (lacidipine)	2
Siofor (metformin)	12
Isoptin (verapamil)	0
Prestarium (perindopril)	21
Valsacor (valsartan)	0
Bisocard (bisoprolol)	31
Areplex (clopidogrel)	3
Tritace (ramipril)	1
Enarenal (enalapril)	0
Dilzem (diltiazem)	3
Metocard (metoprolol tartarate)	3
Diaprel (glicazide)	6
Atrox (atorvastatin)	1
Simvacard (simvastatin)	11
Romazic (rosuvastatin)	2
Others	4
	**Sum (n = 400)**
Knowledge the term of the “OTC”	
Yes	185 (46.25%)
No	215 (53.75%)
Frequency of buying “over-the-counter” medicines	
More than once a week	3 (0.75%)
Once a week	7 (1.75%)
2–3 times a month	41 (10.25%)
Once a month	100 (25.00%)
Less than once a month	246 (61.5%)
No	3 (0.75%)
Place to buy “over-the-counter” medicine outside of the pharmacy	
Liquor store	16
Grocery store	132
Post office	3
Supermarket	139
Drugstore	121
Petrol station	48
Newsstand	28
Internet	44
Only in the pharmacy	79

**Table 2:** Characteristics of responders in terms of study with regression.

						Intensity of Purchasing OTC Drugs outside the Pharmacy	
	Sum (n = 400)					Sum (n = 400)	
	Yes	Rather Yes	I Don’t Know	Rather No	No	Regression	*p*-Value
Do the following factors affect the choice of over-the-counter medicines outside the pharmacy?							
Low prices	135 (33.75%)	85 (21.25%)	61 (15.25%)	49 (12.25%)	70 (17.50%)	−0.117	0.123
Urgency	270 (67.50%)	58 (14.50%)	47 (11.75%)	9 (2.25%)	16 (4.00%)	0.200	0.132
Longer opening of a given store/station than a pharmacy	159 (39.75%)	72 (18.00%)	71 (17.75%)	42 (10.50%)	56 (14.00%)	0.112	0.223
“Saving time” (when shopping)	168 (42.00%)	88 (22.00%)	66 (16.50%)	32 (8.00%)	46 (11.50%)	0.277	0.005
Do you have any awareness of the following factors arising from purchasing drugs outside the pharmacy?							
The lack of pharmacist’s consultation/advice	305 (76.25%)	49 (12.25%)	24 (6.00%)	19 (4.75%)	3 (0.75%)	0.329	0.007
Improper storage	192 (48.00%)	86 (21.50%)	52 (13.00%)	47 (11.75%)	23 (5.75%)	−0.238	0.004
Risk of overdose	189 (47.25%)	66 (16.50%)	48 (12.00%)	66 (16.50%)	31 (7.75%)	−0.189	0.013
Risk of purchasing out-of-date drugs	171 (42.75%)	69 (17.25%)	52 (13.00%	65 (16.25%)	43 (10.75%)	−0.145	0.047
Risk of purchasing falsified drugs	139 (34.75%)	52 (13.00%)	88 (22.00%)	62 (15.50%)	59 (14.75%)	−0.159	0.027
Do you think you need to consult a pharmacist on a given topic?							
Dosage of “OTC” drugs	161 (40.25%)	68 (17.00%)	80 (20.00%)	57 (14.25%)	34 (8.50%)	−0.105	0.170
Side effects resulting from using OTC drugs	159 (39.75%)	85 (21.25%)	74 (18.50%)	48 (12.00%)	34 (8.50%)	−0.062	0.431
Interactions of OTC drugs with medicines used in the treatment of chronic diseases	217 (54.25%)	71 (17.75%)	78 (19.50%)	19 (4.75%)	15 (3.75%)	−0.141	0.131
Storage of OTC drugs	139 (34.75%)	68 (17.00%)	93 (23.25%)	70 (17.50%)	30 (7.50%)	−0.114	0.147
Drug–food interactions	162 (40.50%)	85 (21.25%)	88 (22.00%)	44 (11.00%)	21 (5.25%)	−0.190	0.025
Drugs with dietary supplements interactions	162 (40.50%)	87 (21.75%)	88 (22.00%)	43 (10.75%)	20 (5.00%)	−0.131	0.125
Do you think that using medicinal products simultaneously has impact on the health?							
Aspiryna + Prestarium = Renal failure	105 (26.25%)	38 (9.50%)	233 (58.25%)	9 (2.25%)	15 (3.75%)	−0.199	0.058
Aspiryna + Ibuprom = Prolonged bleeding	163 (40.75%)	51 (12.75%)	160 (40.00%)	14 (3.50%)	12 (3.00%)	−0.015	0.882
Aspirin + Areplex = Prolonged bleeding	121 (30.25%)	43 (10.75%)	220 (55.00%)	9 (2.25%)	7 (1.75%)	−0.080	0.466
Aspirin + Bisocard = Hypetension	117 (29.25%)	44 (11.00%)	209 (52.25%)	11 (2.75%)	19 (4.75%)	−0.164	0.099
Aspirin + Furosemid = Renal failure	120 (30.00%)	48 (12.00%)	204 (51.00%)	13 (3.25%)	15 (3.75%)	−0.078	0.442
Ethanol + Apap = Liver damage	228 (57.00%)	40 (10.00%)	118 (29.50%)	5 (1.25%)	9 (2.25%)	0.090	0.413
Aspirin + Apap = Prolonged bleeding	153 (38.25%)	44 (11.00%)	170 (42.50%)	15 (3.75%)	18 (4.50%)	0.009	0.922
Ibuprom + Areplex = Prolonged bleeding	113 (28.25%)	35 (8.75%)	227 (56.75%)	12 (3.00%)	13 (3.25%)	−0.041	0.684
Ibuprom + Furosemid = Renal failure	121 (30.25%)	34 (8.50%)	219 (54.75%)	13 (3.25%)	13 (3.25%)	−0.044	0.661
Ibuprom + Captorpil = Hypertension	106 (26.50%)	37 (9.25%)	268 (67.00%)	11 (2.75%)	15 (3.75%)	−0.016	0.877
Aspirin + Siofor = Hypoglycemia	107 (26.75%)	34 (8.50%)	239 (59.75%)	7 (1.75%)	13 (3.25%)	−0.084	0.418
Do you think the following symptoms of overdose of OTC drugs that are available outside the pharmacy affect the health of the human body significantly?							
Headaches and dizziness (Aspirin, Ibuprom)	140 (35.00%)	109 (27.25%)	102 (25.50%)	42 (10.50%)	7 (1.75%)	0.074	0.456
Enlargement of the liver (Apap, Gripex)	127 (31.75%)	83 (20.75%)	139 (34.75%)	12 (3.00%)	9 (2.15%)	0.137	0.196
Jaundice (Apap, Gripex)	146 (36.50%)	65 (16.25%)	147 (36.75%)	23 (5.75%)	19 (4.75%)	0.071	0.448
Indigestion, stomachache, nausea and vomiting (All of them)	149 (37.25%)	112 (28.00%)	117 (29.25%)	16 (4.00%)	6 (1.50%)	0.110	0.312
Breathing difficulties (Aspirin)	160 (40.00%)	67 (16.75%)	144 (36.00%)	17 (4.25%)	12 (3.00%)	0.088	0.368
Heart failure (Coffepirine)	161 (40.25%)	65 (16.25%)	149 (37.25%)	15 (3.75%)	10 (2.50%)	0.020	0.839
What are the proposed solutions you believe will contribute to reducing or eliminating the threat of drugs from the non-pharmacy market?							
Reducing dose	97 (24.25%)	65 (16.25%)	72 (18.00%)	103 (25.75%)	63 (15.75%)	0.042	0.573
Presence of a pharmacist in every drug distribution center	140 (35.00%)	82 (20.50%)	72 (18.00%)	69 (17.25%)	37 (9.25%)	−0.020	0.798
Reducing the amount of tablets in the package	73 (18.25%)	56 (14.00%)	70 (17.50%)	117 (29.25%)	84 (21.00%)	−0.077	0.326
Introducing a ban on the sale of drugs to minors and those under the influence	180 (45.00%)	86 (21.50%)	52 (13.00%)	46 (11.50%)	36 (9.00%)	−0.049	0.531
Introduction of licenses for the distribution of medicines without a prescription	198 (49.50%)	88 (22.00%)	62 (15.50%)	29 (7.25%)	23 (5.75%)	−0.013	0.882
Regular inspections conducted by the Main Pharmaceutical Inspectorate and tightening of penalties	217 (54.25%)	97 (24.25%)	57 (14.25%)	20 (5.00%)	9 (2.25%)	−0.057	0.580
Removal of non-pharmacy trade	153 (38.25%)	51 (12.75%)	81 (20.25%)	70 (17.50%)	45 (11.25%)	−0.196	0.007
Medications should only be available in pharmacies	159 (39.75%)	54 (13.50%)	61 (15.25%)	62 (15.50%)	64 (16.00%)	−0.239	0.001

## Data Availability

The exact data can be obtained from the corresponding author.
